# Pregnancy outcome of “delayed start” GnRH antagonist protocol versus GnRH antagonist protocol in poor responders: A clinical trial study

**DOI:** 10.29252/ijrm.15.4.231

**Published:** 2017-04-10

**Authors:** Abbas Aflatoonian, Robabe Hosseinisadat, Ramesh Baradaran, Maryam Farid Mojtahedi

**Affiliations:** 1 *Research and Clinical Center for Infertility, Reproductive Sciences Institute, Shahid Sadoughi University of Medical Sciences, Yazd, Iran.*; 2 *Department of Obstetrics and Gynecology, School of Medicine, Kerman University of Medical Sciences, Kerman, Iran.*; 3 *Department of Obstetrics and Gynecology, Endocrinology and Female Infertility Unit, Roointan Arash Women’s Health Research and Educational Hospital, Tehran University of Medical Sciences, Tehran, Iran.*

**Keywords:** Pregnancy outcome, Poor responder, In vitro fertilization, GnRH antagonist protocol

## Abstract

**Background:**

Management of poor-responding patients is still major challenge in assisted reproductive techniques (ART). Delayed-start GnRH antagonist protocol is recommended to these patients, but little is known in this regards.

**Objective:**

The goal of this study was assessment of delayed-start GnRH antagonist protocol in poor responders, and in vitro fertilization (IVF) outcomes.

**Materials and Methods:**

This randomized clinical trial included sixty infertile women with Bologna criteria for ovarian poor responders who were candidate for IVF. In case group (n=30), delayed-start GnRH antagonist protocol administered estrogen priming followed by early follicular-phase GnRH antagonist treatment for 7 days before ovarian stimulation with gonadotropin. Control group (n=30) treated with estrogen priming antagonist protocol. Finally, endometrial thickness, the rates of oocytes maturation, , embryo formation, and pregnancy were compared between two groups.

**Results:**

Rates of implantation, chemical, clinical, and ongoing pregnancy in delayed-start cycles were higher although was not statistically significant. Endometrial thickness was significantly higher in case group. There were no statistically significant differences in the rates of oocyte maturation, embryo formation, and IVF outcomes between two groups.

**Conclusion:**

There is no significant difference between delayed-start GnRH antagonist protocol versus GnRH antagonist protocol.

## Introduction

Some women undergoing infertility treatments are poor responders to the routine controlled ovarian hyper- stimulation (COH) protocols (1, 2). The ‘‘poor responder’’ was initially defined by Garcia *et al* in 1981 (3). Despite great advances in assisted reproductive technologies (ART) has been established, but the management of poor-responder patients is still a major challenge (4). In IVF programs, the incidence of poor ovarian response (POR) after ovarian stimulation is variable from 9-25% (5). Cause of poor response can be related to age, endometriosis, genetics factors, obesity, or may be iatrogenic such as surgery, radio, and chemotherapy (6, 7). 

Recently, due to changing social structure and the worldwide trend of delaying marriage and childbirth, there has been increased interest in improving the reproductive ability of older women (6). There are no certain definitions of poor response. Recently, the European Society for Human Reproduction and Embryology (ESHRE) has established a new standardized definition for poor responders that called “Bologna criteria” to help identify of these challenging patients for clinical trials and optimal treatment management (8). According to the ESHRE agreement, at least two of the following three features indication must be present: 

1. Advanced maternal age (≥40) or any other risk factor for poor ovarian response,

2. Previous poor response (cycles cancelled or ≤3 oocytes with a conventional stimulation protocol), 

3. Abnormal ovarian reserve test (ORT) (AMH <0.5-1.1 ng/mL or AFC <5-7 follicles). 

In the absence of above criteria, two previous incidents of poor ovarian response after maximal stimulation are sufficient to define a poor responder (8).

Various factors have been associated with a poor response. Alterations in intra ovarian factors or gonadotropin receptor regulation could contribute to suboptimal response (9, 10). Poor responses may result from a shortened follicular phase with limited ability to recruit a follicular cohort or from different sensitivity of early antral follicles to FSH due to follicular different developmental stages with various FSH receptor levels leading to heterogeneity of antral follicle (11, 12). During the last days of the menstrual cycle, FSH increases to preserve antral follicles from atresia and ensure their next growth step (13). Depending on antral follicles inherent sensitivity to FSH, some of them, specially larger follicles are able to respond to the lower levels of FSH better than others, and to start their maturity during the late luteal phase, and leading to asynchronous growth during the first days of the subsequent cycle with COH (14, 15, 16). This lack of coordination in size causes fewer follicles respond to COH (17). 

COH protocols for poor responders are designed to limited early follicle selection in the luteal phase and optimize the follicular hormonal environment and antral follicle responsiveness (14, 16). Oral contraception pills (OCPs) or gonadotropin-releasing hormone agonist (GnRH agonist) long protocol in the late luteal phase can suppress FSH and premature dominant follicle selection. For poor responders, GnRH agonist long protocol or OCPs before GnRH antagonist may cause over suppression of ovarian function and desensitization of the ovary, leading to reduction in the number of mature oocytes and increase the dose of gonadotropins (18-20). 

Administration of luteal estradiol (E_2_) to GnRH antagonist protocols resulted in a reduction of both antral follicular sizes and heterogeneity in the early follicular phase, and increases the number of follicles due to FSH suppression (21-23). Another treatment for these patients is late luteal or early follicular GnRH antagonist administration that suppresses FSH levels and reduces baseline antral follicular size and heterogeneity (24). Recently pretreatment E_2_ and start of antagonist in early follicular phase from day 2-8 before gonadotropin therapy (double suppression) appears to improve ovarian response during COH and may result in more uniform follicular development. This protocol named delayed-start protocol (25). There is no sufficient research from efficiency of current protocol (delayed-start protocol), thus we planned a study about the effect of delayed-start protocol with GnRH antagonist in outcome of ART cycle in poor responders**.**

The objective of this study was to assess the effect of delayed-start GnRH antagonist protocol versus GnRH antagonist protocol in ovarian poor responders.

## Materials and methods

This randomized clinical trial was performed in Yazd Research and Clinical Center for Infertility between March and September 2015. Totally, 60 infertile women between 18-45 yr old with Bologna criteria for ovarian poor responders were allocated in this study. Women with history of endocrine disorders, severe endometriosis, and azoospermia in their husband were excluded. Women were allocated randomly in two groups (delayed and control) according to computer-generated randomization method. 

Control group (n=30) treated with estrogen priming antagonist protocol and case group (n=30) with delayed-start GnRH antagonist protocol. In both group 4mg estradiol valerate tablet (E_2_) (Aburaihan Co., Tehran, Iran) was administered from day 21 in previous cycle and continues for 10 days. In delayed group immediately after administration of E_2_, patients received GnRH antagonist cetrotide (0.25 mg cetrorelix acetate; Merck Serono,Germany) subcutaneously for 7 days, and then we initiated ovarian stimulation with 375 IU FSH (Gonal-f; Merck Serono, Germany).

In control group immediately after administration of E_2_, ovarian stimulation with 375 IU FSH (Gonal-f; Merck Serono, Germany) was performed. In both groups when follicle size was 12 mm, cetrotide added again to prevent premature ovulation and continued until the hCG trigger. When at least two follicles achieved 17 mm in diameter, Human chorionic gonadotropin (hCG) (Choriomon 10000 IU, IBSA Institute, Switzerland) was administered for final oocyte maturation. Oocyte retrieval performed under transvaginal ultrasound guidance 34-36 hr after hCG triggering. Intra-cytoplasmic sperm injection performed with mature oocytes (metaphase II [MII]) in all cycles. 

Day 2 after oocyte retrieval embryos were categorized in four grades from A (high quality) to D (low quality) depending on the number of blastomeres, fragmentation, multinucleation and symmetry; and were transferred with COOK catheter (COOK catheter, USA)(26). 

The main primary outcomes measured were total and mature (MII) oocytes number collected after E_2_ priming antagonist protocol versus delayed-start ovarian stimulation protocol. Secondary outcomes were oocyte maturity rate (MII number /total oocytes number), oocyte yield (total oocytes number /antral follicle count [AFC]), mature oocyte yield (MII number/AFC), total dosage of gonadotropin, ovarian stimulation days, and fertilization rate (two-pronuclear [2PN]/ MII, 16 hr after Intra-cytoplasmic sperm injection treatment). Other secondary outcomes were assessed based on positive serum ßhCG test (chemical pregnancy), 14 days after embryo transfer and observation of gestational sac on transvaginal ultrasound examination (clinical pregnancy), 3 wk after positive serum βhCG.

Implantation rate was defined by the number of gestational sacs divided on the number of transferred embryos in each group. The Ongoing pregnancy rate was assessed as the presence of fetal heart activity by ultrasound after 12 wk. The miscarriage rate was the number of miscarriages before 20 weeks gestation per number of women with a positive clinical pregnancy.

**Ethical consideration**

Our study proposal was approved by Ethics Committee Shahid Sadoughi University of Medical Sciences, Yazd, Iran. Informed written consent was obtained from all couples.

**Statistical analysis**

Data was analyzed using Statistical Package for the Social Sciences 20.0 (SPSS, SPSS Inc, Chicago, Illinois). Continuous data were presented as mean±SD and assessed by independent Student’s *t*-test. Enumeration data were compared by chi-square or Fisher exact test. A P-value<0.05 was considered statistically significant.

## Results

**Figure 1:**
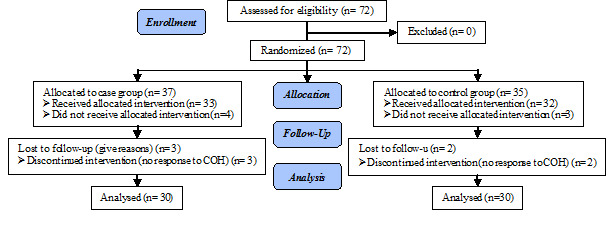
Consort flow diagram

Totally, 72 poor responder women entered to study. 12 women were excluded and finally data of 60 women were analyzed (Figure 1). Baseline characteristics of the patients are presented in table I. The mean age of participants was 38.76±3.46 in cases and 40.30±3.01 in controls, however this difference was not statistically significant. There was no significant difference in Infertility duration, type of infertility, basal FSH level, anti-Mullerian hormone (AMH), antral follicle count (AFC), and previous retrieval cycles between two studied groups (Table I). 

HCG day estradiol and progesterone, gonadotropins dose, and days of ovarian stimulation were similar between two groups (Table II). Endometrial thickness in triggering day was significantly higher in delayed group compared to those of control group (p=0.04) (Table II). There was no significant difference in the number of total and mature (MII) oocyte, obtained and transferred embryos between two studied groups, although there was lower mean in case group versus control group. There were no significant differences in the maturation rate (MII/total oocytes), oocyte yield (Oocytes/AFC), mature oocyte yield (MII/AFC), fertilization rate (2PN/MII) between two groups (Table III).

Although it was not statistically significant difference between two groups in ART outcomes but in delayed-start protocol cycles, chemical (13.30% vs. 3.30%), clinical (13.30% vs. 3.30%) and ongoing (6.66% vs. 3.33%) pregnancy rate and implantation rate (11% vs. 3%) was higher than other group (Table IV).

**Table I.** Baseline characteristics of study participants in both groups

**Variable**		**Case group (n=30)**	**Control group (n=30)**	**p-value**
Age (years)		38.76 ± 3.46	40.30 ± 3.01	0.07
Infertility duration (years)		6.15 ± 4.77	6.70 ± 6.60	0.60
Infertility type				0.19
	Primary	19 (63.3%)	13 (43.3%)	
	Secondary	11 (36.7%)	17 (56.7%)	
Baseline FSH (IU/L)		8.05 ± 2.17	7.76 ± 2.12	0.59
AMH (ng/ml)		0.78 ± 0.49	0.92 ± 0.57	0.34
AFC		4.86 ± 2.09	4.76 ± 2.43	0.86
Previous COH cycle		0.83 ± 0.87	0.63 ± 0.88	0.38

FSH=follicle-stimulating hormone; AMH: Anti-Mullerian hormone; AFC: Antral Follicle Count; COH: Controlled Ovarian Hyper stimulation. Continuous data presented as mean ± SD with p-values obtained from Independent- Samples *t* test; Enumeration data presented as n (%) with p-value obtained from Chi-Square or fisher exact tests.

**Table II:** Cycle characteristics in case and control groups

**Variable**	**Case group (n=30)**	**Control group (n=30)**	**p-value**
hCG day Estradiol (pg/ml)	1152.26 ± 667.55	1233.80 ± 780.64	0.66
hCG day Progesterone (pg/ml)	0.81 ± 0.61	0.58 ± 0.43	0.11
hCG day endometrial thickness (mm)	10.18 ± 2.08	9.13 ± 1.84	0.04
Days of ovarian stimulation	11.60 ± 2.5	12.76 ± 1.50	0.87
Gonadotropin dose (IU)	3372.50 ± 1055.24	3617.50 ± 759.42	0.30

hCG: human Chorionic Gonadotropin. Continuous data presented as mean ± SD with p-values obtained from Independent- Samples *t* test;

**Table III:** Cycle outcomes of study participants in both groups

**Variable**		**Delayed group (n=30)**	**Control group (n=30)**	**p-value**
Total oocytes number		3.63 ± 3.02	5.06 ± 4.37	0.14
MII Oocytes number		2.86 ± 2.50	4.33 ± 3.72	0.07
Maturation rate (( %		77	85	0.29
Oocyte yield		0.86 ± 0.81	1.11 ± 0.87	0.27
MII oocyte yield		0.66 ± 0.60	0.94 ± 0.78	0.13
2PN دumber		1.63 ± 1.67	2.66 ± 2.61	0.10
Fertilization rate ((%		55	62%	0.48
Embryos دumber		1.40 ± 1.56	2.13 ± 1.92	0.07
Transferred embryos number		1.13 ± 1.04	1.56 ± 0.89	0.09
Transferred Embryos quality				
	A	2 (5.88%)	5 (10.63%)	0.91
	B	19 (56.0%)	28 (59.57%)	
	C	11 (32.35%)	12 (25.53%)	
	D	2 (5.88%)	2 (4.25%)	

MII oocyte: metaphase II oocyte; 2PN = Tow pronuclei; AFC: Antral Follicle Count. Continuous data presented as mean ± SD with p-values obtained from Independent- Samples *t *test; Enumeration data presented as n (%) with p-value obtained from Chi-Square or fisher exact tests

**Table IV:** IVF outcomes in case and control groups

**Variable**	**Case group (n=30)**	**Control group (n=30)**	**p-value** *
Chemical pregnancy n(%)	4 (13.30%)	1 (3.30%)	0.17
Clinical pregnancy (%)	4 (13.30%)	1 (3.30%)	0.17
Ongoing pregnancy n(%)	2 (6.66%)	1 (3.33%)	0.35
Implantation rate (%)	11.4%	3.8%	0.27
Miscarriage rate n(%)	2 (50%)	0 (0.0%)	0.36

Enumeration data presented as N (%) with p-value obtained from Chi-Square or Fisher exact tests.

* Chi-Square or fisher exact test.

## Discussion

Despite frequent developments in assisted reproduction, there is no agreement on the effective stimulation protocol for poor ovarian responder patients. In the present study, we compared ART outcomes in poor responders with early follicular GnRH antagonists pretreatment for 7 days after preceding late luteal estrogen priming and before the beginning of ovarian stimulation (delayed start protocol) with GnRH antagonists with estrogen priming without GnRH antagonists pretreatment. Our results showed delayed protocol in poor responders can improve pregnancy and implantation rate although number of oocyte and embryo was lower in this group. Endometrial thickness was significantly higher in study group. It was showed no significant differences in other parameters and ART outcomes.

The definition and treatment of patients with poor response to controlled ovarian hyper stimulation remains controversial. The heterogeneity in patients and inclusion criteria has increased the difficulty in comparing outcomes between the various treatment approaches that have been suggested by different investigators (26). One alternative approach introduced in the1980, was oocyte donation (27). While oocyte donation has become a highly successful option with greater than 50% live birth rate for poor responders most patients are anxious to achieve a pregnancy with others oocytes. Therefore some protocols suggested improving ART outcomes in poor responders. Managing poor response cycles, however, continues major challenges for the reproductive medicines. For more than one decade, GnRH antagonists have been available in IVF preparation. GnRH antagonists prevent premature LH surge without early suppression of follicular development (28).

Pu and colleagues in a meta-analysis compared the use of GnRH agonist protocols with GnRH antagonists of 14 prospective randomized controlled trials. Their result showed no significant difference in IVF outcomes (29). Several approaches have been proposed and investigated to improve poor responder’s treatment outcomes with GnRH antagonists. During the late luteal phase, FSH levels increase progressively to antral follicles ensures growth. Larger follicles are more sensitive to rising levels of FSH and therefore begin to develop during the late luteal phase (30, 31). This discrepancy is detrimental in COH and confused synchronized maturation of the follicular cohort. Coordination of the early antral follicles has been improved by two methods, late luteal estradiol and late luteal or early follicular administration of a GnRH antagonist (26).

Fanchin *et al* in 2003 defined the use of luteal E_2_ to decrease the premature gradual exposure of follicles to FSH in the late luteal phase. By using the late luteal E_2_, there was a significant reduction of mean follicular size at baseline and improvement in overall follicular size coordination (22, 23). In addition, Fanchin et al in another study used one dosage (3mg) of GnRH antagonist in the late luteal phase (on day 25) in normal responders and described that it decreased the exposure of early antral follicles to gradient levels of FSH and synchronized follicular size on day 2 of the cycle pretreated with GnRH antagonist (24).

In another study, Dragisic *et al* demonstrated that the further suppression with either luteal E_2_ patch and 3 days luteal-phase GnRH antagonist appears to be a new option in the treatment of poor responders and yielded superior results compared to patients’ prior IVF cycles (32). However, studies of Fanchin *et al* and Dragisic *et al* were not randomized controls trial. Weitzman *et al* in 2009 demonstrates that the use of E_2_ patch and 3 days GnRH antagonist during the preceding luteal phase in patients with poor history can provide IVF outcomes similar to the microdose GnRH agonist protocol (26).

Ata *et al* in 2011 found similar results in IVF outcomes between luteal E_2_/GnRH antagonists until starting menstruation, and microdose GnRH agonist flare protocol (33). In our study, suppression with GnRH antagonist in early follicular phase was greater than two previous above studies (7 days compare with 3 days) also poor responders inclusion criteria were different. In above studies, E_2_ and GnRH antagonists administered together but in our study GnRH antagonists started after E_2_ priming. Shastri *et al* described that in young poor responders who treated with a luteal E_2_/3 days GnRH antagonist (E2/ANT) protocol, IVF outcomes improved versus an OCP microdose leuprolide protocol (OCP-MDL) (34). In this study mean age of patients was 32 and E_2_ and GnRH antagonist administered together in late luteal phase but in our study mean age were 38. 

Mashayekhi *et al* in 2013 compared the mild antagonist and microdose GnRH agonist flare protocols on IVF outcome in poor responders. they administrated clomiphene citrate before gonadotropin in mild protocol. Endometrial thickness, number of retrieved oocytes, mature oocytes and implantation rate were significantly higher in mild antagonist protocol. Clomiphene citrate improved outcome in antagonist protocol. But in our study suppression before gonadotropin administration improved outcome (35).

Cakmak *et al* demonstrated that the delayed-start protocol (10 days estrogen in late luteal phase then early follicular-phase GnRH antagonist for 7 days before COH) improves ovarian response and IVF outcomes in poor responders compared with E_2_ pretreatment protocol. They showed that double suppression was more effectively from E_2_ suppression alone (25). Cakmak *et al* study showed in E_2_ pretreatment group, ovarian stimulation with gonadotropins was started on cycle day 2 of menstruation after administration E_2_. In case group GnRH antagonists also started on cycle day 2 of menstrual cycle after E_2_, but in our study COH in control group and GnRH antagonists in study group started immediately after completion of the E_2_. Therefore, suppression was shorter in our study.

## Conclusion

In summary based on this study, we concluded that delayed-start protocol in poor responders slightly but no significantly improves pregnancy and implantation rate. Moreover, delayed-start protocol should be investigated in larger prospective randomized studies. Also evaluation of delayed-start protocol without E_2_ priming can compare with other poor protocols such as microdose GnRH agonist flare protocol. According to previous studies (that administrated 3 dose GnRH antagonists before COH) and for reduce costs and treatment duration, it is suggested to design a study for evaluation of lower administration days GnRH antagonist before COH.

## Note

***Registration ID in IRCT:*** ***IRCT2015100424335N1***
