# Corrigendum to “Pregnancy outcome of “delayed start” GnRH antagonist protocol versus GnRH antagonist protocol in poor responders: A clinical trial study” [Int J Reprod BioMed 2017; 15: 231-238]

**DOI:** 10.18502/ijrm.v19i8.9624

**Published:** 2021-09-09

**Authors:** Abbas Aflatoonian, Robabe Hosseinisadat, Ramesh Baradaran, Maryam Farid Mojtahedi

The authors have been informed of some errors that occurred in the published paper. The errors are listed as:

• In the M&M section in the main text, the word “performed” has been changed to “recruited”.
• Some references have been modified in the text.
• The type of randomization has been corrected as the random number table.
• The Mann-Whitney test was added in the statistical analysis section.
• The statistical test which has been used in Table I was added as a table subtitle.
• The study results were re-analyzed, and some p-values were modified in the text and table, especially in Table IV.

The authors wish to apologize for these errors.

The online version of the article has been updated on September 4, 2021 and can be found at http://journals.ssu.ac.ir/ijrmnew/article-1-813-en.html&sw=(http://doi.org/10.29252/ijrm.15.4.231).

